# The Impact of EFL Students’ Emotioncy Level on Their Motivation and Academic Achievement: A Theoretical Conceptual Analysis

**DOI:** 10.3389/fpsyg.2021.798564

**Published:** 2021-12-24

**Authors:** Xuena Zhang

**Affiliations:** College of Foreign Languages, Henan Institute of Science and Technology, Xinxiang, China

**Keywords:** emotioncy level, motivation, academic achievement, positive psychology, positive and negative emotion

## Abstract

Recently, teachers’ and language learners’ emotional status has received special attention among researchers. They argued that learners’ emotioncy level might affect every aspect of language teaching process; therefore, the present study reviewed the impact of EFL students’ emotioncy level on their motivation and academic achievement. Reviewing the literature revealed that there are different classifications for learners’ emotioncy level. However, studies showed that the most prominent classification of learners’ feeling is positive or negative. No matter positive or negative, the main responsibility of teachers in these situations is finding an appropriate way to control these feeling. The paper concludes with some pedagogical implications to control emotioncy in the classroom.

## Introduction

In the learning process, learners are the key participants, and to improve this process, one must pay attention to them and their characteristics (Makiabadi et al., 2019). One of these characteristics that can have an important impact on learning is the feelings and emotions of language learners. Obviously, effective education is not a matter of providing information to a group of students. This type of education is mostly aimed at initiating behavior change in individual students (Datu, 2018).

Many researchers have argued that the main emphasis of previous theories of learning and teaching was on the acquisition of knowledge and skills and ignored complex but definite aspects of human learning, such as the effect of emotional variables on learning, and contextual learning (Aragão, 2011; Berweger et al., 2021). In addition, it is clear that students learn better in dynamic social learning environments where a variety of factors constantly influence each other. As a result, they change the learning environment as well as their own assessment of it (Stockinger et al., 2021). It seems that this feature needs more attention in educational settings. For this reason, a good teacher is one who can increase or decrease that emotion based on awareness of the impact that each emotion has on learning, with proper planning and appropriate educational design (Chang, 2021). Learning theories that focus exclusively on the information process are theories that cannot comprehend this complexity. Researchers believe that these theories have many drawbacks. One of the drawbacks of extensive research on motivation is that, despite the fundamental development and proliferation of theories and models, inactive motivations of individuals are largely ignored. This type of motivation involves constantly thinking about specific concepts or activities, such as spending time doing a good activity without making any serious effort to implement them (Makiabadi et al., 2019; Berweger et al., 2021). Therefore, empirical studies have been developed to examine the effect of emotional variables on learning and performance (Kiuru et al., 2020). Such variables include beliefs about emotions, moods, motivation, and academic achievement. The results of these studies are slowly being incorporated into learning and teaching theories.

One of the notions closely related to learners’ emotions is their motivation. Have you ever wondered why some people have a high level of motivation to progress and work hard to compete with others for success, while others have little motivation to progress and do not strive for success? (Kuklick and Lindner, 2021). Motivation is an inherent phenomenon that is influenced by four factors, namely, position (environment and external stimuli), temperament (internal state and condition of the organism), goal (goal of behavior, purpose, and tendency), and tools (means of achieving the goal; Liu et al., 2021). Humans gain the motivation to achieve their goals, needs, and instincts. For science seekers and students, motivation for academic achievement is of particular importance. With this motivation, people pursue the necessary mobility to successfully complete a task, achieve a goal, or achieve a certain degree of competence in their work so that they can finally achieve the necessary success in learning and academic achievement (Xie and Derakhshan, 2021). Therefore, it can be said that motivation shows the reasons for learners’ behavior and determines why they act in a certain way. Motivated behavior is energy behavior, directional and trailing (Loderer et al., 2020).

From an educational perspective, motivation is a multifaceted structure that is related to learning and academic achievement (Shao et al., 2020). There are many differences and perceptions of motivation. In the field of education, motivation is a three-dimensional phenomenon that includes a person’s beliefs about the ability to perform the activity, the reasons or goals of the person to perform the activity, and the emotional reaction associated with the activity (Seong and Chang, 2021). Experts have divided motivation into two main groups, namely internal and external motivation. Intrinsic motivation creates the necessary attraction to perform an activity, while the person under the influence of external motivation engages in a particular activity with an independent goal (Kuklick and Lindner, 2021). Psychologists have noted the need to pay attention to motivation in education because of its effective relationship with new learning, skills, strategies, and behaviors, and one of the primary structures they have proposed to explain this motivation is motivation for academic achievement (Dörnyei and Ushioda, 2011). Motivation for academic achievement refers to behaviors that lead to learning and progress. In other words, the motivation for academic achievement is the pervasive tendency to do something well in a particular field and to evaluate its performance spontaneously (Imai, 2010).

Many studies have pointed to the significant relationship between achievement motivation and academic achievement and have introduced the existence of achievement motivation as the driving force of academic achievement (MacIntyre and Gregersen, 2012; Swain, 2013). For example, Berkovich and Eyal (2015) in their research have pointed to the effect of motivation on learners’ performance and academic achievement. Academic achievement refers to an individual’s learned or acquired ability in school subjects, which is measured by standardized learning tests or teacher-made tests. In general, this term means the amount of individual learning in the school, so that they can be studied in the general category of factors related to individual differences and factors related to the school and the education system (Burić, 2019). In fact, learners’ academic achievement is one of the most important criteria for evaluating teachers’ performance. For students, the grade point average represents their scientific abilities to enter the world of work and employment and higher educational levels; therefore, educational theorists have focused much of their research on recognizing the factors affecting academic achievement (Cowie, 2011).

Also, since the existence of knowledgeable, efficient, and creative people is an important condition for the growth and prosperity of any society, cultivating and strengthening the motivation for progress causes the creation of energy and proper direction of behavior, interests, and needs of people in line with valuable and specific goals (Houser and Waldbuesser, 2017). In fact, achievement motivation is considered an important factor in academic performance. Motivation to progress as a relatively constant field in students is one of the most important motivations or acquired needs of each individual (King and Chen, 2019). Because learners have different levels of emotion, and these differences affect learning, academic achievement, achievement motivation, thinking, problem-solving, responding to situations, etc., teachers need to be aware of how to help their learners and provide the necessary information (Reeve, 2013).

Since students’ emotion and motivation are among the psychological constructs that play an effective role in students’ academic achievement in various fields and can be a guide in determining their field of study at university and choosing a job in the future, it seems that such research is essential (Reeve and Lee, 2014). What the researcher intends to do in the present study is to highlight the neglected aspect of motivation that was invented as passive motivation. Probably, this hidden motivation is rooted not only in individual differences, but also in the inequalities structured by Bourdieu (1977), the concept of habit, which includes habits and tendencies formed by the experiences of daily life. To confirm the action element, the researcher relied on sensory engagement derived from the emotioncy model, which is a new emotion-based classification for the integration of the senses (Pishghadam et al., 2013).

## Review of Literature

### Emotioncy and Its Levels

**Figure 1:**
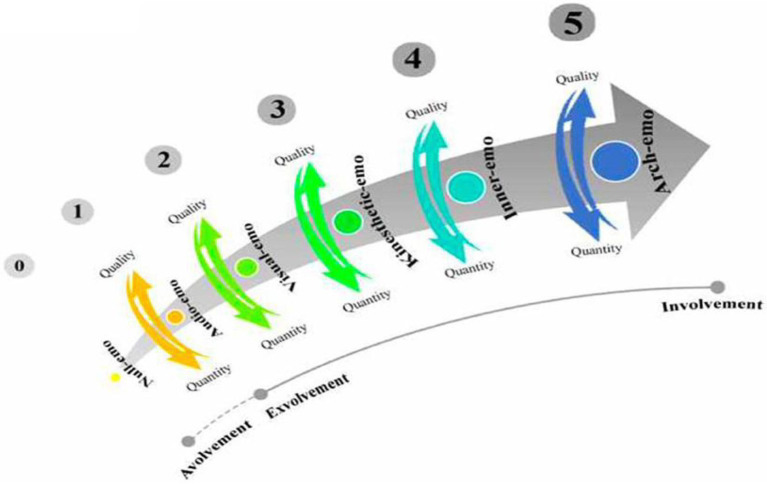
Emotioncy matrix. Adapted from Pishghadam (2015, p. 35).

According to Pishghadam et al. (2013), emotioncy is a combination of senses that provide opportunities for learning. As a relative cognition, sensory experiences evoke emotions through the senses. The emotions and sensory inputs influence learners’ understanding in different ways. According to what Pishghadam and Abbasnejad (2017) stated, emotioncy has six levels that are classified into three main categories. Based on this notion, emotioncy level starts with avolvement (null emotioncy), continues to exvolvement (audio emotioncy, visual emotioncy, kinesthetic emotioncy), and involvement (inner emotioncy and arch emotioncy), which includes the avolvement and exvolvement types. In this sense, each emotioncy level adds to its previous level (Miri and Pishghadam, 2021; See Figure 1).

In this model, emotioncy level starts with avolvement (null emotioncy), continues to exvolvement (audio emotioncy, visual emotioncy, and kinesthetic emotioncy), and involvement (inner emotioncy and arch emotioncy), which includes the avolvement and exvolvement types. In this sense, each emotioncy level adds to its previous level. As the table demonstrates, each emotioncy level builds upon the previous level and includes the features of the preceding emotioncy kind (Pishghadam et al., 2013). The emotioncy notion presents that individuals can construct their idiosyncratic understanding of the world through their senses. L2 learners with more experience of the world in terms of senses involved would have a higher emotioncy level. Consequently, the emotions and the sensory inputs people receive from the environment influence their understanding of reality and perception of the future (Pishghadam et al., 2019). Therefore, it can be argued that people are constantly evolving by the different senses, emotions and feelings they experience. The close relationship between emotions and feelings makes it very difficult to separate the two concepts. In the field of psychology, the inseparable components are emotion, emotion, and mood, while in the educational literature, emotions and feelings are interchangeable and seem close in meaning (Rodrigo-Ruiz, 2016). Emotions are individuals’ natural responses to external events that occur in the body at a certain speed due to external and internal stimuli (Sun and Leithwood, 2015). Emotions are predictable stimuli and can be objectively measured by blood flow, brain activity, hormone secretion, facial features, or body posture (Karami et al., 2019). Emotions are a mental construct that reflect the meanings and analyze a person feeling consciously or unconsciously. Emotions are abstract experiences of feelings; that is, they occur in a part of the mind and are often unique to each person (Tyng et al., 2017). Emotions are often persistent and develop over time. Examples of emotions are joy, sadness, fear, anger, surprise, and hatred (Yin and Lee, 2012).

In EFL contexts, learning in a successful setting is enhanced and exposed to social pressure and social evaluations. Therefore, learning activities may stimulate specific interests and emotions. Feelings and emotions experienced in the classroom context can be positive (e.g., joy, excitement, and pride), or negative (e.g., anxiety, anger, and depression; Zhu et al., 2010; Wang and Guan, 2020; Wang et al., 2021). Indeed, there is ample evidence for the effect of anxiety on learning; if the effect of emotions, such as anger, happiness, or depression, on learning and action, is less known. This lack of attention may be due to the lack of clear theoretical frameworks before the 1980s, and the complexity of feelings and emotions’ measurement (Dewaele and MacIntyre, 2014). In the 1980s, several researchers attempted to break down emotional experiences into their separate components and attribute a central role to the evaluation process. For example, Tsai (2012) argues that emotions are accumulated in memory along with verbal and practical knowledge. This information may serve as a cleanser to warn of future and present events and to be used when difficult and non-difficult situations are identified.

Swain (2013) explains that emotions are not present in a situation, but rather in a personal assessment of an incident. In other words, events are related to the inner expression that satisfies (non-problematic, favorable situation, related to perception and emotions); or they are annoying (problematic, threatening situations that can lead to destruction, damage, or loss). This means that increased physiological arousal (e.g., increased muscle and muscle tension, heart rate, and transpiration) changes the readiness for activity, but that unique interpretation of arousal and the event that caused it is the nature of emotion. It determines its impact on action (Méndez, 2011).

In educational settings, students may get angry for a variety of reasons; for example, when they are questioned by a teacher, or when they are not allowed to do their favorite job. Such situations can increase the level of physiological evaluation in most students (Pishghadam, 2009). In addition, some students reported milder anger and resentment, while others expressed extreme anger. Hsu (2010) tried to learn more about the various situations that make students upset in class, and concluded that elementary and high school (ages 10–14) are the situations that cause the most anger.

In the last decade, the role of emotions in education has become more prominent and is considered an important role in learning (Pishghadam et al., 2017). The learners’ emotions seem to affect learning by engaging the learner and their attitude toward learning and the learning environment (Houston, 2016; Pishghadam et al., 2020). Emotions are very important in learning and affect learning performance. According to psychologists and neuroscientists, emotions play an essential role in cognitive learning, and the performance of cognitive activities is enhanced by positive emotions. Emotions affect the learner’s ability to process information and to accurately understand what he or she is experiencing (Wang et al., 2021). Emotions are generally classified as pleasant (positive) and unpleasant (negative). Emotions can affect learning both positively and negatively. Often when the learner experiences positive emotions, the learning process can be improved and when the learner experiences negative emotions, the learning process can fail (Fathi et al., 2020; Wang and Guan, 2020). Swain (2013) classifies positive emotions into four categories: happiness, interest, satisfaction, and love, and believes that positive emotions expand the scope of attention and increase insight and creativity. While positive experiences give people a better chance to learn, grow and develop, negative emotions prevent them from learning. Students in the positive state have higher levels of motivation than those in the neutral state.

Many researchers believe that EFL motivation is emotionally driven (Dent and Koenka, 2016). Paying attention to the emotions in the language teaching process can overcome the problems of apathy that are caused due to various reasons, such as fear or anger, which can jeopardize the potential of foreign learners’ development (Derakhshan et al., 2020b). Besides, trying to evoke emotions in language teaching contexts might increase students’ self-esteem, self-confidence, and empathy that might boost students’ motivational energy and facilitate language learning (Burić, 2019; Chang, 2021). The different emotions that EFL teachers and learners may experience during learning activities can cause various affective reactions in EFL teachers and students. These reactions are a set point for their future activities (Gordeeva et al., 2019).

Strengthening positive psychology in the classroom helps to build a positive identity in students. Everyone involved in the training cycle can have an impact on identity formation. Teachers, principals, parents, classmates are among the influential people in this process. These effects can be positive or negative. Other factors, such as educational system, culture and educational environment, textbooks, and students’ personality, can determine different levels of emotion, motivation to learn as well as their academic achievement. Makiabadi et al. (2019) investigated the role of emotioncy in Willingness to Communicate. The findings of the study confirmed that emotional, cognitive, and sociocultural emotioncy have a significant impact on language learners’ performance and their Willingness to Communicate. The results showed that there was a statistically significant positive relationship between emotioncy and willingness to write (WTW), willingness to read (WTR), willingness to speak (WTS), and willingness to listen (WTL). Besides, the results revealed that among these three types the cognitive type is a positive predictor of WTR and WTL. Seong and Chang (2021) examined the relationship between emotioncy and learners’ burnout and achievement in a sample of 450 South Korean adolescents. The results of this study demonstrated that positive emotions and negative emotions negatively and positively predicted academic burnout and achievement, respectively. Stockinger et al. (2021) focused on the same concept but in a different mode of instruction. They investigated the difference between traditional teaching contexts and online mode of instruction and the changes in emotions, motivations, and academic achievement. The findings of the study showed that a new mode of instruction negatively affected emotions and increased learners’ anxiety. However, it increased their hope and adaptability that will lead to learners’ better performance. Shao et al. (2020) analyzed the interaction between emotion and EFL performance. They found that there is a positive relationship between control-value appraisals and learners’ emotions and academic achievement. They recommended that teachers need to know and consider learners’ perceptions of emotion and language performance.

Kuklick and Lindner (2021) investigated computer-based immediate Knowledge of Results (KR) feedback and its impact on learners’ motivation and emotions. They argued that feedback has a different and fluctuating impact on learners’ motivation and emotions. Individual differences, cultural and contextual factors are among the factors that might influence learners’ motivations and emotions. In a similar study, Loderer et al. (2020) analyzed 186 research studies that investigate changes in emotions in TBLEs that were published between 1965 and 2018. They found that there is a significant relationship between emotions, such as enjoyment, curiosity/interest, anxiety, anger/frustration, confusion, and boredom and their antecedents that are control-value appraisals, prior knowledge, gender, and TBLE characteristics and learners’ outcomes that can be engagement, learning strategies, and achievement.

### Motivation

Motivation for progress is one of the psychological issues that has attracted the attention of many psychologists and education specialists. Motivation is in fact the main motivator and energy that causes behavior (Berweger et al., 2021; Pishghadam et al., 2021) and if the effective factors in creating academic motivation are known and used properly, significant successes in the process of education and learning and most importantly continuity of learning will be created. The motivation for progress is the power to do good things to the highest standards. Due to the effect of academic achievement motivation on student success, in recent decades, psychologists have sought to study and identify the factors affecting academic achievement motivation. Their research findings showed that personality, family, academic, and social variables are related to this structure (Mahdavi et al., 2021).

Demotivation is a situation in which students believe that norms, laws, or rights have been violated and that there is no plausible excuse (Derakhshan, 2021). However, in the classroom, it is always possible for anger to be directed freely to the source of provocation and anger, and learners must learn to suppress their anger in order to stay in school. Intense and persistent anger often indicates symptoms of behavioral problems. In addition, suppressing and controlling anger puts a lot of strain on a person’s processing power and may hinder performance. Moreover, research in mental health (psychology) shows that anger in the evaluation system can be a threat to health (Fathi et al., 2021). The effects of depression (sadness), stress, happiness, and happiness in the classroom environment have not been widely studied. In addition, it can be assumed that increases in the level of arousal labeled sadness and happiness may evoke perceptions and emotions that conflict with information processing capacity (Derakhshan et al., 2020a). In contemporary psychology, there is evidence that proves the effect of positive and negative moods and moods on perceptual flow. Researchers conducted numerous studies that show that situations that cause a particular mood can affect the information processing system. They stated that those who have a positive mood want to express positive experiences and emphasize the positive aspects of the text (Borsipour et al., 2019). They spend a lot of time coding information that suits their mood and remember the last more positive things about a text. In the case of a negative mood, they do the opposite. They also describe studies in which positive and negative moods affect performance perceptions themselves and influence problem-solving and decision-making processes. These studies suggest that emotions and moods alert students to whether or not the environment in which they work is problematic, in order to regulate the flow of information according to the state of the environment (Derakhshan et al., 2021).

Working on positive psychology, Liu et al. (2021) found that students’ independent motivation mediates the relationship between perceived teacher support and creative self-efficacy. They also found that students’ successful feelings (enjoyment and relaxation) mediated the relationship between perceived teacher support and creative self-efficacy, but the mediating effects of negative success feelings (anxiety and boredom) were not significant. Chang (2021) explored various dimensions of emotions and their impact on learners’ motivational engagement and academic performance. By focusing on cognitive test anxiety, the study found that emotions are strong predictors of academic performance by increasing learners’ motivational engagement. In a similar study, Datu (2018) demonstrated that various dimensions of emotions have been constantly associated with maladaptive psychological, physical health, and educational achievement. The study showed that there is a strong indirect relationship between negative emotions and learners’ engagement and performance.

#### The Dual Continuum Model of Motivation

**Figure 2:**
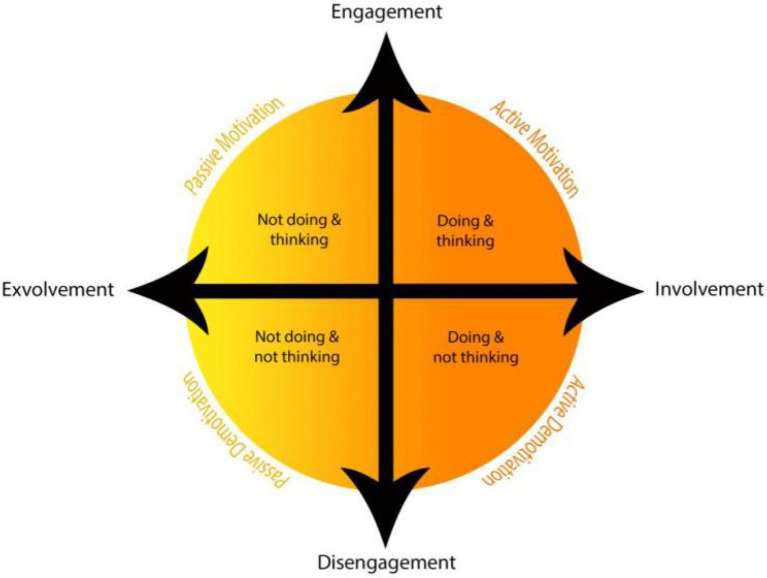
The dual continuum model of motivation. Adapted from Pishghadam (2019, p. 20).

In order to develop a more comprehensive view of motivation on the basis of immersion (i.e., action and cognition) and to address its active and passive dimensions, Pishghadam et al. (2019) proposed a dual continuum model with engagement as one continuum, and involvement as a separate one (See Figure 2).

In psychology, engagement is regarded as an approach relevant to intrinsic and extrinsic motivations (Calder and Malthouse, 2008). Within the realm of education, engagement accounts for positive emotions, boosting hope, motivation, and optimism in students (Pekrun et al., 2011). As a result, they become more engaged in studying and general education (Ouweneel et al., 2011). Involvement is closely associated with behavioral engagement (Macey and Schneider, 2008). However, as Pishghadam (2015) suggests, engagement may be different in nature depending on the number of senses which are put into play. From this standpoint, involvement, with its sensory nature, rests upon the psycho-linguistic concept of emotioncy, which is believed to exert considerable influence on motivation.

Engagement and involvement are two interrelated, yet distinct, constructs which have been tied to thinking (mental activity) and doing (physical activity), respectively. Presence or absence of engagement (i.e., engagement and disengagement) interacted with various degrees of sensory involvement (i.e., exvolvement and involvement) divides the model into two halves (i.e., active and passive) and four slices (comprising active motivation, active demotivation, passive motivation, and passive demotivation). As its ideal form, active motivation is when an individual is fully engaged and involved in performing a task. Lack of adequate mental engagement, however, alters this optimal performance to a mechanical behavior (active demotivation). Passive motivation explains the condition in which individuals do not get the opportunity of turning thoughts or motivational preferences into action, yet keep pondering over the issue constantly. Passive demotivation, as the least active condition, represents no specific cognitive or physical activity (Pishghadam, 2016; Pishghadam et al., 2016).

### Academic Achievement

As one of the most significant gauge of the effectiveness of education, the academic achievement of learners can be influenced by various factors, such as teaching methods, teaching materials, and learners’ motivations (Yin and Lee, 2012). One of the criteria for the performance of any educational system is learners’ academic achievement. Academic achievement is the capability to demonstrate academic success in attaining intended conclusions. Many researchers highlight the impact of mental and cognitive abilities on learners’ academic achievement (Rodrigo-Ruiz, 2016; Greenier et al., 2021).

However, having a high level of emotioncy does not guarantee academic achievement and EFL teachers should be aware of their learners’ emotional status. Learners’ emotional status might affect acquiring, analyzing and internalizing newly acquired knowledge (Derakhshan et al., 2019). Kiuru et al. (2020) investigated the relationships between motivation, emotion, and task performance. They found that the high value of the work, high hopes for success, and high positive emotions before doing a work contribute to a higher level of effort during the work. They also found that expecting great success predicted an increase in positive emotions throughout the work. Conversely, high negative emotions while doing work are related to poorer performance. And finally, they argued that high task performance was associated with higher levels of effort, greater success-to-ability ratio, and increased positive emotions after work. Both job performance and attribution of success were linked to subsequent better academic achievement. Berweger et al. (2021) argued that according to Pekrun’s control-value theory motivation and emotion are essential elements in learners’ academic engagement, learning in the classroom, and achievement-related outcomes.

In the last decade, the role of emotions in education has become more prominent, and they play an important role in learning. The learners’ emotions seem to affect learning by engaging the learner and their attitude toward learning and learning environment. Emotions are very important in learning and affect learning performance. According to psychologists and neuroscientists, emotions play an essential role in cognitive learning, and the performance of cognitive activities is enhanced by positive emotions. Emotions affect the learner’s ability to process information and to accurately understand what he or she is experiencing (Seong and Chang, 2021).

Emotions are generally classified as pleasant (positive) and unpleasant (negative). Emotions can affect learning both positively and negatively. Often when the learner experiences positive emotions, the learning process can be improved and when the learner experiences negative emotions, the learning process can fail. To improve the quality of learning, teachers need to pay attention to the learners’ emotions (Aragão, 2011). Emotion control skill is one of the most important skills that a person can use to effectively manage or respond to an emotional event. Humans subconsciously use emotion regulation strategies to overcome difficult situations (Berkovich and Eyal, 2015).

Students experience different emotions in everyday life. The main emotions of human beings include sadness, anger, fear, joy, surprise, shame, and jealousy. A student who can control his emotions can use his skills in appropriate situations. As a result, he will not feel pressured or forced to suppress them (Houser and Waldbuesser, 2017). For example, if a person correctly recognizes that he or she is angry right now, he or she can identify the trigger for anger, then make better decisions about what is right. Students who do not have the ability to regulate emotion feel stressed because of their emotions. They may have to do things that keep them from feeling bad, but each of these behaviors can be dangerous or lead to harm. Lack of emotion control skills can also have adverse effects on students’ social relationships. Students who have no control over their emotions may just suppress them and eventually become anxious and depressed because of the repressed emotions. Learning and practicing emotion control skills is not like taking medicine for a set amount of time. Our lives are in a bed of current emotions so we are constantly experiencing them. If you want to control your emotions, you have to be constantly trying. Similar to the findings of Kiuru et al. (2020), negative emotions in the workplace are categorized as relative and in the form of spectrum. Negative emotions include Frustration/resentment, Worry/stress, anger, Hatred and disgust, and Annoyance. There are negative and positive emotions in everyone. It is good for humans to be able to manage their negative emotions and replace their positive emotions with practice. Students face a variety of emotions in their daily lives. In other words, they have some emotions with them from the moment of birth, and this shows the importance and role of different emotions in life. Some emotions are pleasurable, pleasant, and invigorating, so that one may struggle to reach that particular state of emotion.

## Conclusion

This study reviewed EFL students’ emotioncy level, their motivation, and academic achievement. Reviewing the literature, the researcher found that there was a relationship between the components of motivation and academic achievement and with the results of researchers who found a significant and high relationship between motivation to gain dignity, power, encouragement, ability, competitiveness, social dependence, achieving future goals, interest in learning and acquisition. The satisfaction of others with the continuation of education and the progress of students’ education was emphasized, it is somewhat consistent. The studies emphasized the importance of combining the senses and emotions in determining learners’ preferences and learning styles.

Cognitive control refers to cognitive processes, such as emotions (e.g., evaluations and self-belief) or special interests in a subject. As explained, cognitive control, which has high intrinsic motivation and high self-efficacy, is intended to psychological behaviors, which initiate or continue the activity in a skillful manner, lead to and affect the quality and quantity of effort. In addition, the important point is that in a learning subject, many behavioral intentions have been formed, some of which have been established, while others are not according to the student’s wishes and decisions.

Datu (2018) noted that behavioral law is difficult to learn, especially in the face of attractive behavioral options, or when social pressure undermines a commitment and responsibility. In such cases, students must actively divert their attention from competing for action tendencies and maintain their learning intentions. This active method of control is called action bias and is in contrast to the passive control method called state bias. People who score high on “state bias” focus more on their emotional state than on homework. Another difference between students who have no control over action is: (a) the inability to start work due to hesitation and hesitation, (b) the inability to continue the work due to lack of continuous interest, (c) Inability to continue the desired work due to intellectual preoccupation to failure. Pishghadam et al. (2013) called this relative cognition process “emotioncy.” They argued that a combination of the senses and emotions are eminent factors in determining learners’ specific preferences and learning styles.

### Implications

The first implication is that EFL students need to acquire how to manage their emotions. Emotion management skills are one of the most important skills students need when dealing with their classmates and teachers. If a student is unable to control his or her emotions, he or she may engage in aggressive or depressive behaviors and significantly reduce his or her self-esteem and self-esteem. The most important actions that EFL teachers can do to increase students’ emotion management skills are the following:

*Introducing emotions and the senses:* First, familiarize the student with your different types of inner emotions and its relationship with their senses. Explain to him that the feeling of anger, happiness, fear, disgust, surprise, and sadness is a kind of psychological-behavioral reaction that occurs naturally in the human body to deal with certain situations and situations.

*How to interact with emotions and the senses:* The student must be fully convinced that suppressing or expressing emotions completely is not a good way to control and interact with one’s inner emotions and the senses. Since the presence of emotion to a certain extent within learners motivates and promotes in various fields, it is better to control it and use it well instead of coping.

*Check the level of excitement:* Talk to the students about the emotion they have just experienced. Ask them out well if they are no longer absorbed in the connection. By examining a number of emotions, estimate the average amount of each emotion together. The combination of the senses and emotions should be taken into account to define learners’ specific emotion in learning contexts.

*Talking about emotional reactions and its relationships with the senses and their behaviors:* Talk to the student about the relationship between emotions and behavior and make it clear that although it is perfectly normal and unconscious for him to have emotions, reacting to these emotions is optional and the student is fully responsible for his or her own reactions. Therefore, the occurrence of a wrong reaction can lead to reprimand, punishment, and exclusion from the community of friends.

*Investigate the excitement created in others:* Just as a particular behavior or situation may cause upset, anger, and other emotions in a person, so the student’s reaction and behavior to his or her inner emotions can evoke the emotions of his or her audience. By examining the outcome of the emotional response to the audience and talking about it, the student can understand the impact of their emotional behavior and remind them of the importance of strengthening emotion management skills. It is easier for the student to understand the feelings of others and the behavior caused by the emotions if the student has a high level of mastery of empathy skills.

*Determine the best response to emotions:* Now that the student is fully aware of his or her natural physiological emotions and is aware of the extent of the reaction and its effect on others, it is time to talk to him or her about the best response to each emotion. Writing down how you feel, being patient, optimistic, understanding the feelings and emotions of others, and learning how to let go of negative emotions are some of the most important steps you can take when the next emotions arise.

The second pedagogical implication relates to multisensory teaching in facilitating learning. Several studies recommended the creation of a sensory-rich environment by adding more sensory cues to the teaching topics and engaging learners in more sensory inputs. Hence, teachers are encouraged to incorporate multiple senses into their teaching practices to help learners increase their level of emotioncy (Shayesteh, 2019; Seyednozadi, 2021).

The third and final implication could be that it is not at times motivating to involve learners in the concepts or activities that they prefer to exvolve or opt out. In order to motivate each learner, teachers need to appreciate the weight of senses either in isolation or in combination and have a sufficient level of emo-sensory intelligence (ESQ). According to Pishghadam and Shayesteh (2017), ESQ deals with the ability to recognize, label, monitor, and manage sense-induced emotions to guide one’s behavior. It is important to keep in mind that learners with high levels of sensory capital may be less reluctant toward sensory motivation, since they have already been involved in distinct sensory experiences.

### Future Studies

Reviewing the literature reveals that many factors, such as individual differences, cultural, and contextual factors, might play a key role in shaping learners’ emotioncy level and further engagement and learners’ achievement. Future studies can focus on different levels of emotioncy in language learning environments. Cross-cultural studies are required to determine culture dependence and context dependence of this notion. Since the notion of emotioncy deals with emotions and the senses, longitudinal studies are preferened to highlight its latent aspects in language teaching contexts over time. Finally, the mode of instruction might play a key role in determining the learners’ emotional status. It is suggested that the future studies consider these factors in online settings.

## Author Contributions

The author confirms being the sole contributor of this work and has approved it for publication.

## Funding

This paper was supported by Provincial Project of Philosophy and Social Sciences of Henan Province in 2021: English Online Learning Engagement and Its Intervention Strategies under Intelligent Learning Environment (grant number 2021BYY008).

## Conflict of Interest

The author declares that the research was conducted in the absence of any commercial or financial relationships that could be construed as a potential conflict of interest.

## Publisher’s Note

All claims expressed in this article are solely those of the authors and do not necessarily represent those of their affiliated organizations, or those of the publisher, the editors and the reviewers. Any product that may be evaluated in this article, or claim that may be made by its manufacturer, is not guaranteed or endorsed by the publisher.
